# Importance and regulation of adult stem cell migration

**DOI:** 10.1111/jcmm.13422

**Published:** 2017-12-07

**Authors:** Beatriz de Lucas, Laura M. Pérez, Beatriz G. Gálvez

**Affiliations:** ^1^ Universidad Europea de Madrid Madrid Spain; ^2^ Instituto de Investigación Hospital 12 de Octubre Madrid Spain

**Keywords:** migration, stem cells, cancer, cytoskeleton

## Abstract

Cell migration is an essential process throughout the life of vertebrates, beginning during embryonic development and continuing throughout adulthood. Stem cells have an inherent ability to migrate, that is as important as their capacity for self‐renewal and differentiation, enabling them to maintain tissue homoeostasis and mediate repair and regeneration. Adult stem cells reside in specific tissue niches, where they remain in a quiescent state until called upon and activated by tissue environmental signals. Cell migration is a highly regulated process that involves the integration of intrinsic signals from the niche and extrinsic factors. Studies using three‐dimensional *in vitro* models have revealed the astonishing plasticity of cells in terms of the migration modes employed in response to changes in the microenvironment. These same properties can, however, be subverted during the development of some pathologies such as cancer. In this review, we describe the response of adult stem cells to migratory stimuli and the mechanisms by which they sense and transduce intracellular signals involved in migratory processes. Understanding the molecular events underlying migration may help develop therapeutic strategies for regenerative medicine and to treat diseases with a cell migration component.

**Table:** 

**• Introduction**
**• Adult stem cell migration in physiological and pathological settings**
**• Regulation of stem cell migration**
**• Mechanisms of cell migration**
**• Conclusions**
**• Acknowledgements**
**• Conflict of interest**

## Introduction

Adult stem cells are rare populations of cells present in almost all adult mammalian tissues and are responsible for tissue‐specific maintenance and repair 1, 2. A foremost hallmark of stem cells is their capacity for asymmetric cell division, wherein one daughter cell gives rise to a committed clone that has the ability to proliferate and differentiate (multipotency capability), while the other daughter cell remains undifferentiated, contributing to the long‐term maintenance of the stem cell pool (self‐renewal capacity).

**Figure 1:**
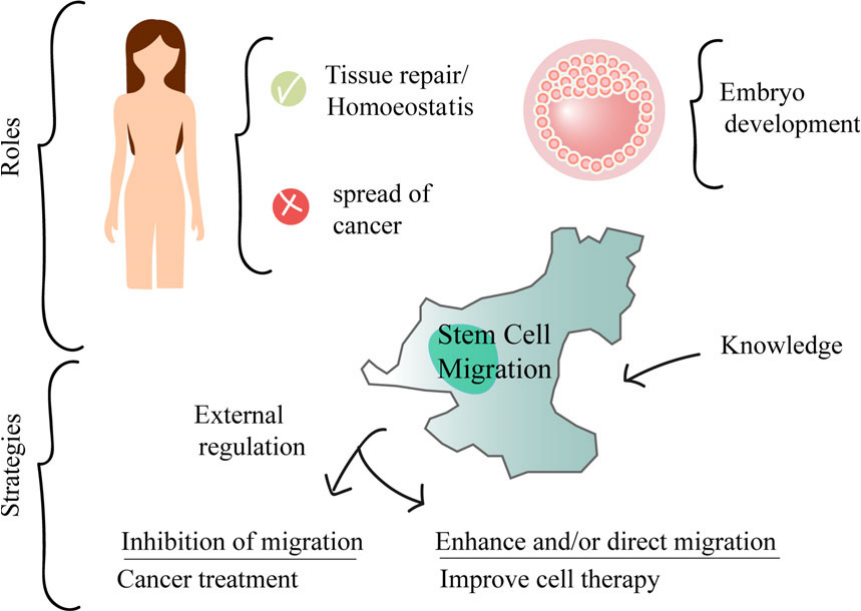
Stem cell migration is required for embryogenesis, for homeostasis and repair of adult tissues, but also plays an important role in the development of cancer. Therefore, migration capacity of stem cells is a fundamental characteristics necessary to carry out their function. The acquisition of knowledge of this process could be potentially used in cell therapy increasing and/or directing migration and in treatments of diseases with cell migration involved inhibiting this migration.

Stem cells also have a natural ability to migrate throughout the life of vertebrates. At the first stages of embryogenesis, stem cells divide, migrate long distances, establish in new locations and specialize 3, 4. Once tissues and organs are formed, adult stems cells remain one of the few cell types that retain the capacity to migrate under appropriate stimuli 5, 6. Thus, stem cell migration is fundamental not only during embryonic development, but also during adult tissue homoeostasis and repair (Fig. 1).

**Figure 2:**
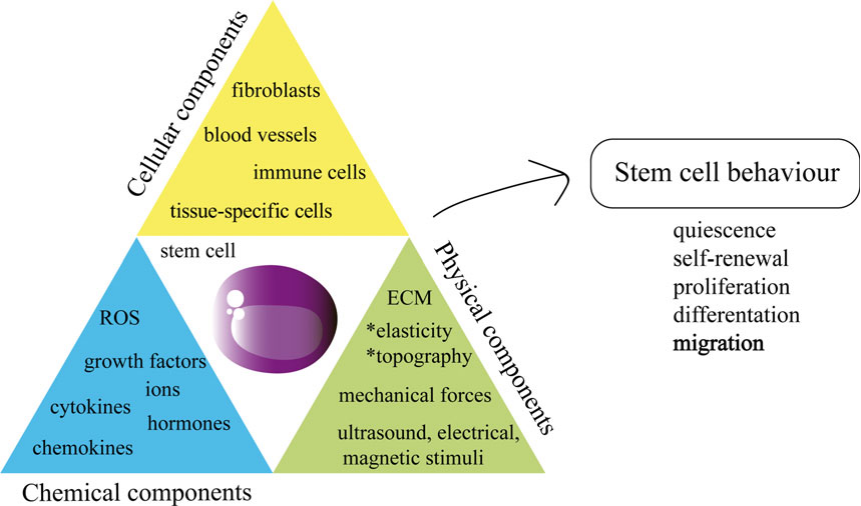
Stem cell behaviour is controlled by the niche which comprises their cellular, physical and chemical components. Cellular components include all the cells types that reside in the niche as blood vessels, immune cells or tissue‐specific cells. Secreted factors such as chemokines, growth factors or cytokines are considered chemical components of the niche. The physical components as extracellular matrix (ECM), mechanical forces are also involved in the regulation of stem cell functions. The quiescence, self‐renewal, proliferation, differentiation and migration of stem cells are governed by the combination of these three components of the niche.

Stem cell behaviour has to be tightly regulated for correct tissue homoeostasis, particularly the balance between self‐renewal and differentiation. To accomplish this, adult stem cells often reside in specific regions or niches, which not only provide structural support, but also regulate stem cell fate by integrating intrinsic and extrinsic factors and local and systemic signals 7 (Fig. 2). The interaction between stem cells and other cell types, such as endothelial and inflammatory cells, extracellular matrix (ECM), soluble factors (growth factor, cytokines, hormones…), physical characteristics (*e.g*. tissue stiffness) and environmental signals (*e.g*. hypoxia), is all included in the term niche 8. Niches also seem to have a close relationship with blood vessels 9, 10, conceivably facilitating the influx of systemic factors and damage signals, and the mobilization of stem cells into circulation.

The stem cell niche needs to be sufficiently dynamic to adapt to injury situations and to the different requirement of the tissues. Adult stem cells are present in the niche in a quiescent, but reversible, undifferentiated and self‐renewable state, retaining their capacity to exit quiescence and proliferate and differentiate on demand 11. The niche therefore provides a protective environment to preserve self‐renewal and prevent the exhaustion of stem cells pools by excessive proliferation 12. It also plays an important role in the decision of a stem cell to migrate 13.

## Adult stem cell migration in physiological and pathological settings

In the following sections, we describe the most characterized adult stem cells and typical migration modes seen in adult tissues. A better knowledge of migration processes has the potential to be used as strategies for cancer therapy and inflammatory diseases, and for cell‐based treatments in regenerative medicine.

The adult stem cell with the most remarkable migratory capacity is undoubtedly the hematopoietic stem cell (HSC). HSCs are multipotent bone marrow (BM) cells that represent 0.01–0.15% of all of nucleated cells and give rise to all cellular components of the blood, including leucocytes, erythrocytes and platelets 14. HSCs can egress from the BM niche into the circulation, extravasate into different tissues and then ingress into BM 15. Under physiological conditions, there is continual flux of HSCs between the blood and BM that is regulated by circadian rhythms 16, 17. Different injury paradigms (haemorrhagic shock, inflammation, stroke, among others) lead to a significant increase in the pool of HSCs in circulation 18, 19, although their contribution to tissue repair and regeneration is unknown. The remarkable ability of HSCs to migrate and return to the BM niche is harnessed in the clinical setting for BM transplantation because allogeneic HSCs also exhibit tropism to recipient BM and give rise to all hematopoietic cells 20.

Mesenchymal stem cells (MSCs) are considered multipotent, non‐HSCs, present in almost every tissue, that can differentiate into several distinct mesenchymal cell types and also into cells from other germ layers 21, 22. It is believed that MSCs resides in perivascular niches 23, and their presence in each tissue could facilitate their migration and arrival at injury sites. The endogenous migration of BM‐MSCs under injury situations has been described, and work has shown that MSCs enter circulation and reach damaged tissues to promote tissue regeneration 24, 25. It has also been documented that some adipose tissue‐derived MSCs can migrate *via* the lymphatic system under conditions of inflammation 26. The migration or homing of administered MSCs in a therapeutic context is clearly of great interest due to their potential for regenerative medicine applications. MSC homing is defined as the transmigration of MSCs across the endothelium after their arrest within the vasculature of a tissue 27 through processes that are well characterized 28, 29. Accordingly, directional migration of MSCs is dependent on chemotactic signals from injured or inflamed tissues and is associated with the expression of the migration/attachment factors CD44 and CXCR4 30, 31. Nevertheless, intravenous infusion of MSCs generally leads to their entrapment in the lung, liver and spleen 32; consequently, a major current focus in the field is to determine strategies to increase MSC homing and survival after infusion, which is challenging within the tissue injury environment.

Endothelial progenitor cells (EPCs) are involved in neovascularization (vasculogenesis) and therefore contribute to *de novo* post‐natal neovascular formation 33. It is proposed that after ischaemic injury, EPCs are mobilized and differentiate, but they can also produce cytokines and growth factors (*e.g*. VEGF, SDF‐1 and IGF‐1) that promote the migration of resident progenitor cells and mature endothelial cells to contribute to neovascularization 14. The important role of EPCs is supported by the finding that their levels are reduced or dysfunctional in some diseases such as diabetes 34, 35, and also by the functional improvement of tissues after EPC transplantation in different disease models 36, 37.

Several mammalian organs remain in a state of flux throughout life, suggesting strong activity of their stem and progenitor cell populations. In contrast to adult stem cells, progenitor cells are more pre‐committed to differentiate into a specific cell type, and their self‐renewal capacity is limited. Progenitor cells can therefore be regarded as an intermediate state between stem cells and the fully differentiated cells 38. In the intestine, epithelial stem cells are localized near the bottom of the intestinal crypts and, during renovation of the epithelia, they proliferate and differentiate into progenitor cells, which mature and migrate until they reach the epithelium 39. As the skin is the first line of defence against environmental assault, it requires continuous regeneration to function correctly 40. Skin stem cells need to leave their niches (at the basal layer of inter‐follicular epidermis, hair follicles and other skin compartments), migrate and differentiate to maintain tissue integrity. With regard to the progenitor cells of the basal layer of the epidermis, it has been described that they first have to detach from the underlying basement membrane and then migrate while at the same time progressively mature until they reach the outermost epidermal skin layer 1. While skeletal muscle is considered a stable organ, it has a great capacity to regenerate after injury situations (*e.g*. extreme exercise, trauma or disease). The best‐studied progenitor cell type of skeletal muscle is the satellite cell (SC), so‐named because it is located surrounding the basal lamina and outside the myofibre plasma membrane. SCs exist in a quiescent state in their niche until injury, after which they are stimulated to activate and proliferate, and subsequently migrate to undergo myogenesis. Once SCs differentiate into mononucleated myoblasts, some of them divide asymmetrically to replenish the SC compartment and to produce mature muscle fibres 41. Bone is a dynamic tissue that undergoes continuous remodelling to maintain its correct structure. This process requires the finely balanced activity of osteoblasts (bone formation cells) and osteoclasts (bone resorption cells). Osteoblasts have a mesenchymal origin 42, whereas osteoclasts arise through differentiation of hematopoietic progenitors, specifically the mononuclear macrophage/monocyte‐lineage 43. Osteoblasts, osteoclasts and stromal cells are necessary for bone remodelling, and chemotaxis and migration are crucial for this process 44. Initially, osteoclasts require activation to perform resorption. Accordingly, monocyte precursors from BM egress to circulation, migrate and differentiate into mature osteoclasts on the bone surface 45. Once bone resorption is complete, osteoblasts migrate to the bone pit to begin the task of bone formation by synthesizing extracellular bone matrix. Subsequently, osteoblasts can differentiate into osteocytes, appearing embedded in the bone matrix, or undergo apoptosis 42.

Migration of stem cells in an uncontrolled manner can lead to pathological situations such as cancer. There is intense debate on the origin of tumours, and it is recognized that great variability exists among the cells that form a tumour. A theory that continues to gain traction is the stem cell theory of cancer, which posits that cancers arise from a few primitive stem cells, termed cancer stem cells (CSCs), which are present in tissues and accumulate all mutations necessary to initiate tumourigenesis 46. There is great similarity between CSCs and normal stem cells, and they share many features and behaviours 47, 48. The biological properties of stem cells support their involvement in the origin of tumours as they transmit genetic material throughout life and conceivably accumulate genetic heritable alterations due to their high division activity during embryonic development and adult homoeostasis. Moreover, their migration capacity can be enhanced by an altered microenvironment. The observation that cancer risk increases with age fits well with a stem cell model whereby stem cells are maintained throughout life and retain their ability to divide, possibly resulting in cumulative damage that would increase the risk of cancer later in life 49. Further, it seems that there is a correlation between the number of stem cell divisions in a given tissue and the risk for developing cancer in that tissue 50.

The stem cell of origin of cancer together with the migratory capacity of CSCs might provide an explanation for situations where the primary tumour is often not identified at autopsy. In this regard, metastasis could be redefined as the formation of a tumour by cells from another tissue and without the requirement that those cells have to originate a tumour in the tissue of origin 49. Metastases are the main cause of many cancer‐related deaths. Because metastasis requires migration, the migratory capacity of tumour cells is a potential target for therapy. As for normal adult stem cells, the regulation of cancer cell migration is a complex process involving many factors such as chemoattractants, ECM and communication with neighbouring cells 51, 52. In this regard, two types of CSC can be distinguished within tumours: stationary or mobile. Stationary CSCs are involved in the growth of tumour, whereas mobile CSCs are involved in dissemination of the tumour 53, 54. Therefore, it is important for successful cancer therapy to consider both stationary and mobile CSCs to not only reduce the size of tumours, but also to eliminate migrating CSCs otherwise the tumour will re‐emerge.

## Regulation of stem cell migration

Dynamism of the cell cytoskeleton allows for the adaption, in space and time, to environmental factors, generating migratory responses. The regulation of migratory responses as well as quiescence, proliferation and differentiation of stem cells is governed by signalling from the niche.

Cells are able to sense and mount a response to physical and mechanical properties and forces. A chief architect of these responses is the ECM, which is involved in many important cell functions including adhesion, migration and differentiation 55, 56. Cells are able to distinguish dimension, orientation, density, stiffness and even elasticity of the ECM, and adapt their behaviour according to the topography of the underlying ECM 57. Because of this, mechanical stimulation by ultrasound 58, 59 or vibration 60, which increase cell migration, is used as non‐invasive techniques for wound healing processes. Other mechanical forces such as static strain can also promote cell migration 5. In addition, electrical stimulation 61 or endogenous electric fields and magnetic stimuli 62, 63 also have effects on cell migration.

Nevertheless, cells are not only affected by their immediate environment. The arrival of soluble factors such as cytokines 64, 65 or growth factors 66, 67, presumably produced in more remote areas, is sufficient to activate receptors and initiate migration signalling. Stem cell migration is also affected by the arrival of hormones of different origins 68. They are able to stimulate the migration and mobilization of the stem cells as happen to HSCs in the present of parathyroid hormone 69 or to MSCs with erythropoietin 70. The question therefore arises, what are the different stimuli that affect cell migration and how do cells sense environmental changes and adjust to these cues? Cells present different receptors that, after their activation by a stimulus, activate the appropriate signalling pathway to migrate in response to environment signals. The presence or the absence of the receptor for each stimulus in different migratory cell types enables the limitation of the input to target cell types.

The process by which cells sense environmental mechanical signals and translate them into biochemical responses is known as mechanotransduction 71. This mechanism requires cell–matrix or cell–cell adhesion in combination with contractility of the actin cytoskeleton to sense and respond to changes in ECM 72.

Cell‐ECM adhesion complexes are multiprotein complexes that serve as sites of attachment between the cell and the surrounding ECM *via* integrin binding, which allows physical connection of the cell actin cytoskeleton with the ECM. Integrins are membrane glycoproteins with three domains, intracellular, transmembrane and extracellular, enabling them to function as linkers of the cell to the ECM, in both directions, through the connection with the cytoskeleton. Contractile forces generated by the interaction of actin and myosin filaments can be transmitted to the ECM substrate, triggering modifications to its surface. Moreover, the cell can sense the topography of the ECM substrate and respond accordingly 72. ECM adhesion complexes, besides acting as sites for adhesion and allowing cells to sense external mechanical forces, also function as traction points that are needed for cell movement 73. Different multiprotein complexes are involved in mechanotransduction 74. Similar to integrins, cadherins are transmembrane proteins that mediate cell–cell contacts by forming adherent junctions between cells, rather than facilitating cell‐ECM contact 75, 76. Moreover, soluble factor can be recognized by cells *via* transmembrane proteins that activate signalling pathways after binding their ligands, such as growth factor, hormone and cytokine receptors. Yet, other ways to sense stimuli could be the glycocalyx 77 or primary cilium (non‐motile cilium) 78, 79.

Cell migration depends upon the transmission of intracellular signals. Although a great variety of signals and receptors affecting migration exist, they all ultimately converge on the same ‘migration pathway’, the RhoA‐ROCK‐myosin II axis 78. Specificity is derived from the nature of the stimulus, how the cell receives the stimulus, the presence of the receptor involved, and the initial steps of signalling. Clearly, migration processes implicate many molecules that are interrelated and have to be precisely regulated.

**Figure 3:**
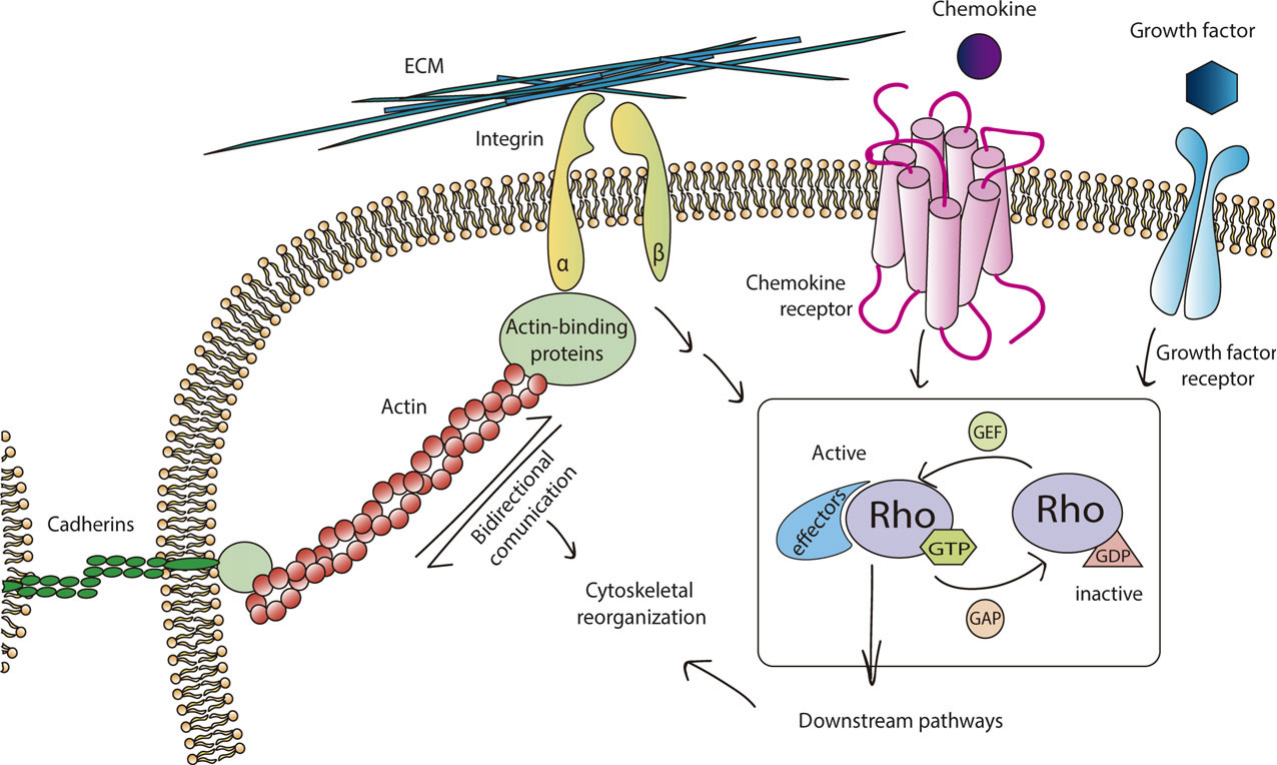
Cells are able to sense a great variety of migratory stimuli using different types of receptors. They have receptors for soluble factors as chemokines and growth factor receptors. To sense environmental mechanical signals, cells have receptors as integrins and cadherins that have the capacity to translate mechanical signals into biochemical responses, knowing this process as mechanotransduction. Once the cell receives the signal (physical, chemical or cellular) different signalling pathways are triggered and converge on the Rho family of small GTPases which are considered as master regulators of actin cytoskeleton reorganization. They are molecular switches by cycling between a GTP‐bound active state (mediated by GEF) and a GDP‐bound inactive state (catalysed by GAP).

Transmembrane receptors such as integrins, cadherins, growth factor receptors and cytokine receptors, can sense tensional forces and change their conformation accordingly following mechanical or physical stimuli or ligand binding (Fig. 3). This engagement/activation generates signalling cascades that converge on the Rho family of small GTPases, especially RhoA, and it regulators, which are powerful modulators of actin cytoskeletal rearrangements 80.

Although chiefly recognized as regulators of the actin cytoskeleton, Rho GTPases also control cell growth, membrane trafficking and transcriptional regulation, among others. Rho GTPases are members of the Ras superfamily of 20–30 kD GTP‐binding proteins that act as molecular switches by cycling between a GTP‐bound active state and a GDP‐bound inactive state 81. Signalling is turned on by guanine nucleotide exchange factors that catalyse nucleotide exchange of GDP for GTP, whereas GTPase‐activating proteins stimulate GTP hydrolysis, leading to inactivation and signalling is turned off. Although the Rho GTPase family comprises 20 members, three ubiquitous members, RhoA, Rac1 and Cdc42, are the more studied. To date, nearly 100 effectors are known for the mammalian Rho family that function spatially and temporally and, as a consequence, have the potential to be therapeutic targets of different diseases that affect migration such as cancer 81, 82.

Briefly Rac1 and Cdc42 regulate actin polymerization; Cdc42 also seems to be fundamental for polarization and chemotaxis as cells migrate randomly in its absence. RhoA controls the assembly of contractile actiomyosin filaments and is responsible for cell contractility. All three proteins regulate microtubule cytoskeleton and gene transcription and are also involved in the formation of matrix adhesion complexes 83. Downstream effectors of RhoGTPases modulate the polymerization, organization and contraction of actin, polymerization and stability of microtubules, and transcriptional regulation of cell motility (motogenic) genes 84.

The RhoA‐ROCK‐myosin II axis is a fundamental migratory pathway. Briefly, RhoA activates Rho kinase (ROCK), which in turn promotes myosin II activity. This results in increased intracellular contractility that is fundamental to maintain the basal tension required for mechanosensing in both directions 85. RhoA, through other effectors, leads to stabilization of actin filaments and promotes actin filament polymerization, which is also related to migration and plays a strategic role in the selection of the mode of cell migration 86. Consequently, the RhoA‐ROCK‐myosin II axis emerges as the common and central hub for the regulation of migration 86, 87, 88.

## Mechanisms of cell migration

Cell migration has been traditionally studied *in vitro* using two‐dimensional (2D) extracellular matrices, permitting easily accessible, convenient and observable experiments. A great innovation in the field in recent years has been the introduction of *in vivo* migration studies and three‐dimensional (3D) *in vitro* models, which have revealed the great diversity and plasticity of migration mechanisms. Indeed, cell behaviour in 2D environments can substantially deviate from behaviour in 3D. While the majority of cells on 2D substrates extend lamellipodia, these projections are more extensive in 3D environments along with the presence of multiple modes of migration, comprising lamellipodia, lobopodia, blebs and collective migration 89. There is no unique form of cell migration. Cells are able to migrate individually or in groups, directionally or randomly. Furthermore, a single cell can display different migratory structures that result in a defined migratory mode and with it specific characteristics 90.

Cell migration can be random or directional. Random migration is considered when cells move randomly with frequent changes in direction. The converse is directed cell migration, where cells migrate in a determinate direction due to a stimulus. Directional migration can be governed by the intrinsic predisposition of cells to continue migrating in the same direction without changing, or by external stimulus 91. The nature of the external stimulus determines the type of movement and its terminology. When cell movement in a specific direction is controlled by the gradient of a soluble factor, it is known as chemotaxis, whereas haptotaxis is associated with a gradient of cellular adhesion sites or substrate‐bound chemoattractants on a surface. Directed movement in an electrical field is termed electrotaxis, whereas movement towards mechanically stiff signals is termed durotaxis 92, 93.

Collective migration consists of coordinated and cooperative migration as cohesive groups of cells instead of as a single cell. In the last decade, collective migration has emerged as a fundamental phenomenon in development as it is involved in the formation of the tissues and their compartments (*e.g*. epithelial cells, endothelial cells and neural crest cells), and it is also related to cancer and metastasis. It is also important to maintain adherens junctions stability between collectively migrating cells 92, which increases the efficiency of paracrine signalling and coordination between cells. If adherens junctions are absent, cells migrate as single entities, but when junction is less stable or intermittent, a coordinate but individual migration of many cells known as *chain migration* or *cell streaming* can occur. These migration modes are inter‐convertible due to the formation or resolution of cell junctions 57.

Cells travel across 3D environments using a surprisingly diverse array of migration modes and inter‐conversion between them, termed migratory plasticity, is frequently observed 94. Furthermore, in some cases distinct migration modes are not mutually exclusive and can be found in the same cell under some conditions 95, 96.

Following the nomenclature of Petrie *et al*. 86, a pseudopodium (which literally means false foot) is defined as any protrusion of the cell that is able to extend and retract. The classification of these protrusions in the different migration modes that exist is based according to the structure of the leading edge. The principal distinguishing migration modes are lamellipodia, filopodia, blebs and lobopodia, although other types such as invadopodia and podosomes are also occasionally observed. The lamellipodium is a protrusion on the leading or seeking edge of the cell and is considered the basis of migration for the majority of motile cells on 2D structures. Lamellipodia are thin sheet‐like extensions of the cytoplasm driven by actin polymerization and integrin adhesion 86. Activation requires Rac proteins that relay signals to WAVE proteins that in turn stimulate the Arp2/3 complex, which is involved in the nucleation and assembly of branched actin filaments 92. Filopodia are finger‐like and actin‐rich extensions at the leading edge and serve to sense the microenvironment. The formation of filopodia is triggered by the Rho GTPase Cdc42 86, 92. Blebs are dynamic cytoskeleton‐regulated plasma membrane protrusions driven by cortical actomyosin contractility. Migration *via* blebs is occasionally described as amoeboid migration as the movement resembles that of rounded amoebae. This type of migration mode has gained great importance as it is observed in 3D environments but rarely in 2D cultures 97, 98. The principal distinctive feature of blebs is that they are mediated by hydrostatic pressure and not by actin polymerization. Blebbing initiates with a breach in the actin cortex or by the detachment of the cortex from the plasma membrane, which allows cytoplasm to push through the breach to form a bleb. An actin cortex is reformed once expansion slows. Blebbing is linked to high levels of RhoA/ROCK signalling 92. Cells that migrate *via* blebbing present integrins localized in a diffuse manner, which can indicate weaker interactions between the cell and the ECM than in other migration modes such as lamellipodial or lobopodial 57. Lobopodia are cylindrical protrusions with lateral small blebs, and consequently have characteristics both of blebs and lamellipodia. Like blebs, lobopodia are driven by intracellular pressure and are linked to Rho/ROCK‐myosin II signalling. In more pliable 3D matrices, fibroblasts use lobopodia and require adhesion to ECM, mediated by integrins, and actomyosin contractility, for correct migration 92. Lobopodia utilize integrin‐dependent adhesion similar to lamellipodia but have more actomyosin contractility and higher intracellular pressure 94. Invadopodia and podosomes are actin‐rich protrusions capable of degrading ECM and infiltrating 3D environments. The term invadopodia is used when these protrusions are on cancer cells and are related to invasion and metastasis, whereas the term podosome is used for non‐transformed cells. The adhesion of these structures is dependent on integrins 86. Cdc42 is the principal Rho GTPase involved in the formation of invadopodia 92. Podosomes can found in different cell types, including endothelial cells, osteoclasts and macrophages 99.

The migration mode can be documented through the combination of the grade of cell–matrix adhesion and actomyosin contractility. The proposed molecular mechanisms that instruct the migration mode in 3D is governed by cell–matrix adhesions, RhoA signalling and actomyosin contractility 86. Microenvironmental changes induce switching between migration modes. Typically, there is a correlation between migration mode and the 3D environment; the nature of the environment frequently determines how a cell will migrate 86.

## Conclusions

Cellular migration is crucial throughout the life‐time of an organism. Cells can migrate in very different ways, randomly or directed (by diverse stimuli), individually or collectively, and display numerous diverse protrusions at the leading edge. Accumulating evidence suggests that RhoA‐ROCK‐myosin II axis is the key pathway not only to sense the microenvironment (mechanotransduction), but also in the selection of the migration mode. Stem cells have a natural ability to migrate that is fundamental for tissue homoeostasis and repair. Thus, a better understanding of this molecular pathway and other related pathways could help in the development of new strategies for regenerative medicine to improve directed cell migration and migration inhibitory treatments against inflammatory diseases and cancer.

## Conflict of interest

The authors confirm that there is no conflict of interests.
